# Assessing Genomic Diversity and Productivity Signatures in Dianzhong Cattle by Whole-Genome Scanning

**DOI:** 10.3389/fgene.2021.719215

**Published:** 2021-10-05

**Authors:** Xianfu Zhang, Kaixing Qu, Peng Jia, Jicai Zhang, Jianyong Liu, Chuzhao Lei, Bizhi Huang

**Affiliations:** ^1^ Key Laboratory of Applied Technology on Green-Eco-Healthy Animal Husbandry of Zhejiang Province, Zhejiang Provincial Engineering Laboratory for Animal Health Inspection and Internet Technology, College of Animal Science and Technology, College of Veterinary Medicine, Zhejiang A&F University, Hangzhou, China; ^2^ Yunnan Academy of Grassland and Animal Science, Kunming, China; ^3^ Key Laboratory of Animal Genetics, Breeding and Reproduction of Shaanxi Province, College of Animal Science and Technology, Northwest A&F University, Xianyang, China

**Keywords:** Dianzhong cattle, hybrid, genetic diversity, selection signatures, DDX4

## Abstract

Dianzhong cattle is a classic Chinese indigenous cattle breed with historical records dating back to 200 BC. But with its genomic differences having not been clearly elucidated, the quest for genomic characterization will be an essential step towards understanding the genomic basis of productivity and adaptation to survival under Chinese farming systems. Here we compared 10 Dianzhong cattle (four newly sequenced and six downloaded) with 29 published genomes of three underlying ancestral populations (Chinese zebu, Indian zebu, and Yanbian cattle) to characterize the genomic variations of Dianzhong cattle. Dianzhong cattle has a high nucleotide diversity (0.0034), second only to Chinese zebu. Together with analyses of linkage disequilibrium decay and runs of homozygosity, Dianzhong cattle displayed higher genomic diversity and weaker artificial selection compared with Yanbian cattle. From a selective sweep analysis by four methods (*F*st, π-ratio, XP-CLR, and XP-EHH), the positive selective signals were mainly manifested in candidate genes and pathways related to heat resistance, growth and development, fat deposition, and male reproduction. Missense mutations were detected in candidate genes, *SDS* (c.944C > A and p.Ala315Glu), *PDGFD* (c.473A > G and p.Lys158Arg), and *DDX4* (rs460251486, rs722912933, and rs517668236), which related to heat resistance, fat deposition, and spermatogenesis, respectively. Our findings unravel, at the genome-wide level, the unique diversity of Dianzhong cattle while emphasizing the opportunities for improvement of livestock productivity in further breeding programs.

## Introduction

Domestic cattle generally refers to two subspecies, *Bos taurus* and *Bos taurus indicus*. They were domesticated in the Fertile Crescent (∼10,000 years ago) and the Indus Valley (∼8,000 years ago), respectively (Utsunomiya et al., 2019). These two subspecies can be interbred without barriers, unlike other bovine subspecies (buffalo, American bison, etc.) that have reproductive isolation (Wu et al., 2018). According to previous genomic study, the domestic cattle in the world could be divided into six major groups: European taurine, Eurasian taurine, East Asian taurine, African taurine, Indian indicine, and Chinese indicine (Chen et al., 2018). Besides that, as one of the important routes for the migration of Indian indicine into China, there are many hybrid cattle with different lineages in the Yunnan region of China.

Dianzhong cattle is one of the most widely distributed indigenous breeds in Yunnan. It has a long history of breeding, with historical records dating back to 200 BC (China National Commission of Animal Genetic Resources, 2011). In addition to agricultural use, it also has social importance, including during marriage, birth, death, and sacrificial ceremonies, as well as being regarded as representatives of wealth, prestige, and status (China National Commission of Animal Genetic Resources, 2011). Due to the hot and humid climate of the original area, Dianzhong cattle display superior heat tolerance and resistance to parasites. Although small in size, its labor performance is excellent, owing to its long-term use as the main farming and transportation livestock (Wen et al., 2014). Moreover, as a classic indigenous cattle breed, it also shows advantageous characteristics of high intramuscular fat, strong disease resistance, and crude feed tolerance (Hao et al., 2017). In recent years, on account of its slow growth and low reproductive performance, which cannot meet the growing needs of beef, local people have blindly and massively introduced commercial cattle for hybrid improvement (Hao et al., 2017). However, blind hybridization as well as the lack of breed conservation planning caused the threat of breed degradation in Dianzhong cattle.

Based on whole-genome sequencing, many studies initially focused on the genetic architecture and economic traits under positive selection in commercial breeds (Bovine et al., 2009; Stothard et al., 2011), and then the focus gradually shifted to the adaptation of indigenous breeds, such as climate tolerance and disease resistance (Kim et al., 2017; Taye et al., 2017; Kim et al., 2020). With the development of next-generation sequencing technology and the enrichment of re-sequencing databases, genome-wide genetic analysis studies play an increasingly powerful role in the investigation of germplasm resources of landraces, such as cold tolerance of Yanbian cattle (Shen et al., 2020), excellent meat quality of Mongolian cattle (Chen et al., 2021), heat tolerance and parasite resistance of African cattle (Kim et al., 2017), and growth rate and feed conversion of Jiaxian red cattle (Xia et al., 2021). An earlier study on Dianzhong cattle using Illumina BovineHD BeadChip (777K) demonstrated that Dianzhong cattle contained mainly indicine ancestry mixed with taurine ancestry. Based on the estimate of observed heterozygosity and expected heterozygosity, Dianzhong cattle had the maximum level of inbreeding coefficients in Yunnan region (Li et al., 2019). In fact, single-nucleotide polymorphism (SNP) array data with only limited and known SNPs might make many important genetic information hard to detect. In order to further explore the genetic potential of Dianzhong cattle, we compared the genome re-sequencing data of 10 Dianzhong cattle (including four that were newly sequenced) with 29 reference cattle from Yanbian, Jiangxi, and India to search for the candidate signatures of positive selection by four methods (*F*st, π-ratio, XP-CLR, and XP-EHH).

## Materials and Methods

### Sample Collection and Sequencing

We sampled four Dianzhong cattle from Chuxiong, Yunnan, China. Genomic DNA was extracted from the ear tissue samples as previously described (Jia et al., 2019). The pair-end libraries were constructed for each individual (500 bp insert size), and the DNA was subjected to Illumina NovaSeq sequencing using 2 × 150 bp model at Novogene Bioinformatics Institute (Beijing, China). Additionally, genomes of 35 publicly available representative groups, including Dianzhong cattle (*n* = 6), Chinese zebu (Jiangxi cattle, *n* = 10), Indian zebu (Brahman, Gir, and Nelore, *n* = 10), and Asian taurine (Yanbian cattle, *n* = 9), were used for the combined analysis (Supplementary Table S1). To obtain clean reads with high quality, the raw data was modified using Trimmomatic (LEADING:20 TRAILING:20 SLIDINGWINDOW:3:15 AVGQUAL:20 MINLEN:35 TOPHRED33) (Bolger et al., 2014).

### Alignments and Variant Identification

Clean reads were aligned against the latest *B. taurus* reference genome (ARS-UCD1.2) using Burrows-Wheeler Aligner BWA-MEM (v0.7.13-r1126) with default parameters (Li et al., 2009). The Picard tool (http://broadinstitute.github.io/picard) was used to filter potential duplicate reads (REMOVE_DUPLICATES = true). SNP calling was performed by Genome Analysis Toolkit 3.8 (GATK) (Nekrutenko et al., 2012; Haplotype Caller, Genotype GVCFs and Select Variants module). After SNP calling, we used the module “Variant Filtration” of GATK to obtain high-quality SNPs with the parameters (“DP < 156 (1/3-fold total sequence depth for all individuals) || DP > 1404 (3-fold of total sequence depth for all individuals) || QD < 2.0 || FS > 60.0 || MQ < 40.0 || MQRankSum < −12.5 || ReadPosRankSum < −8.0 || SOR > 3.0”). Finally, we used ANNOVAR (Wang et al., 2010) to annotate the functions of the SNPs based on the *B. taurus* reference assembly ARS-UCD1.2.

### Population Genetic Diversity

Nucleotide diversity for each group was investigated by VCFtools (Danecek et al., 2011) with a 50 kb non-overlapping window across all autosomes. The genetic relationship among four groups was performed by principal component analysis (PCA). Autosomal SNPs in high levels of pair-wise linkage disequilibrium (LD) were pruned using PLINK (Purcell et al., 2007) with the parameter (--indep-pair-wise 50 5 0.2) and were conducted using the smartPCA program in the EIGENSOFT v5.0 package (Patterson et al., 2006).

The LD decay for each group was measured using PopLDdecay (Zhang et al., 2019) with default parameters. Runs of homozygosity (ROHs) were identified using the Runs of Homozygosity program implemented in PLINK, which slides a window of 50 SNPs (-homozyg-window-snp 50) across the genome in estimating homozygosity (Xia et al., 2021). The following settings were performed for ROH identification: -homozyg-density 50 -homozyg-window-het 1 -homozyg-window-missing 2. The number and length of ROH for each group were estimated, and the length of ROH was divided into three categories: 0.5–1, 1–2, and 2–4 Mb reflecting ancient, historical, and recent inbreeding, respectively (Bhati et al., 2020). The strong linkage disequilibrium between parental genomic loci formed ROH, with long haplotype segments derived from recent ancestors and short haplotype segments derived from earlier ancestors (Jane et al., 2006).

### Selective Sweep Identification

To identify selective sweep regions, genetic differentiation (*F*st), genetic diversity (π-ratio), cross-population composite likelihood ratio test (XP-CLR), and cross-population extended haplotype homozygosity test (XP-EHH) were performed with 50 kb sliding window and 20 kb step between Dianzhong cattle and each other reference group separately. Selection scanning was performed using these four methods based on allele frequency, linkage imbalance, and population differentiation. The overlap of the top 1% windows in each method was considered as candidate signatures of selection. Genomic regions identified by at least two methods were considered to be candidate regions of positive selection (Sun et al., 2020). In addition, π and *F*st were computed with 5 kb sliding window and 2 kb step by using VCFtools for each candidate gene. To gain a better understanding of the gene functions and signaling pathways of the identified candidate genes, online Kyoto Encyclopedia of Genes and Genomes (KEGG) pathway and Gene Ontology (GO) analyses were conducted using DAVID 6.8 (Huang et al., 2009). The analyses for LD and construction of the haplotypes were performed with the online SHEsis software (http://analysis.bio-x.cn/myAnalysis.php) (Yong et al., 2005).

## Results

### Genome Resequencing and SNP Identification

Individual genomes of four Dianzhong cattle were generated to an average of ×∼9.8 coverage each and aligned to the *B. taurus* reference genome ARS-UCD1.2 with an average alignment rate of 99.72%. To reveal the diversity of Dianzhong cattle, 10 Dianzhong cattle (four new and six downloaded) were jointly genotyped with 29 publicly available genomes from three representative groups (10 Indian zebu, 10 Chinese zebu, and nine Yanbian cattle) (Supplementary Table S1). In total, 49,094,970 bi-allelic autosomal SNPs were detected, and the average alignment rate and sequencing depth of the final set reached 99.50% and ×∼12, respectively. After functional annotation of the polymorphic sites, the largest number of SNPs in the exon region was Dianzhong cattle (616,110). That presented the richer genetic diversity of Dianzhong cattle (Supplementary Table S2). Meanwhile, the analyses of unique SNPs and non-synonymous SNPs displayed that the genetic diversity of Dianzhong cattle was second only to Chinese zebu (Supplementary Figure S1).

### Population Genetic Diversity and Relationships

**FIGURE 1:**
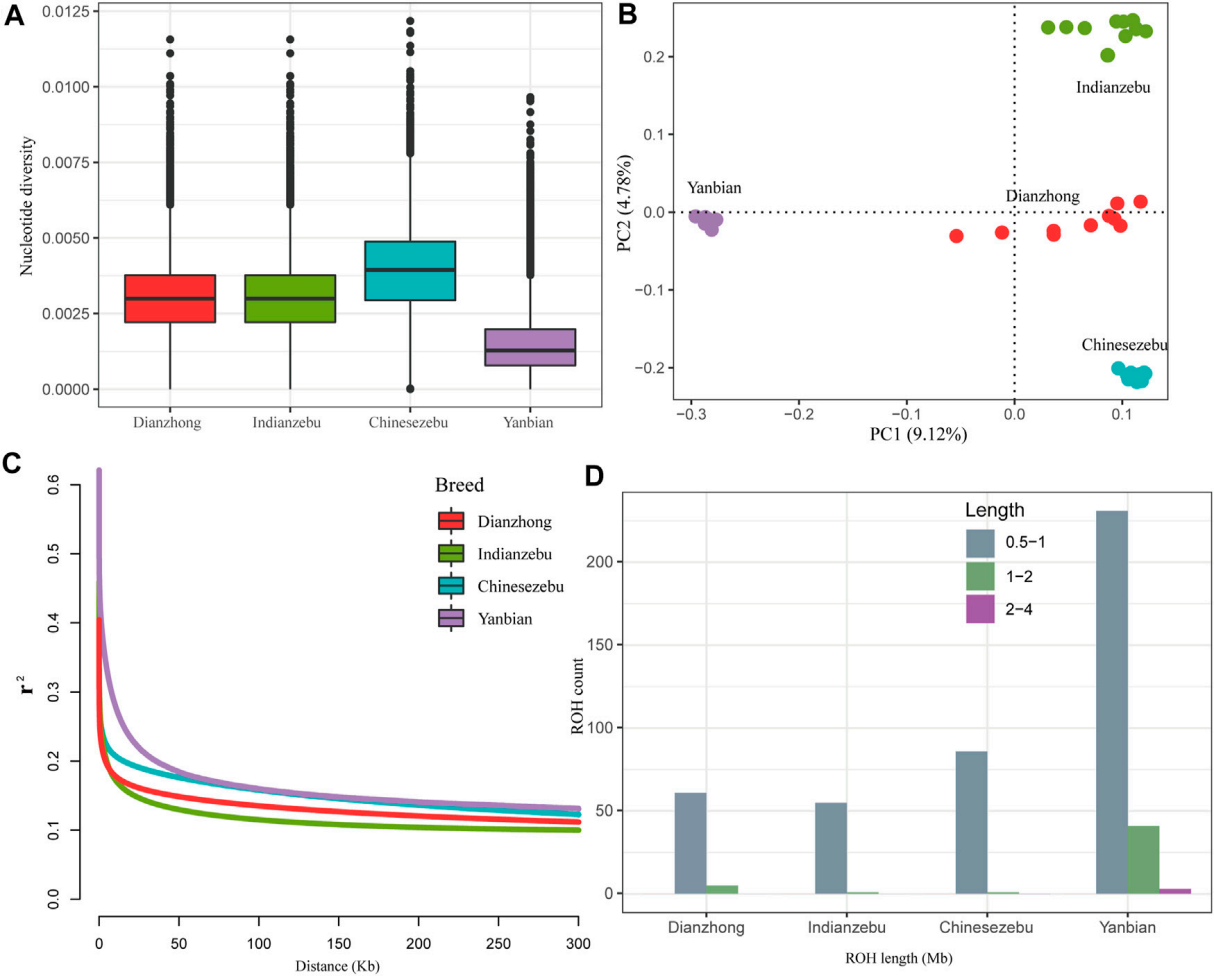
Summary statistics for genomic diversity. **(A)** Genome-wide distribution of nucleotide diversity of each group. The horizontal line inside the box indicated the median of this distribution; box limits indicated the first and the third quartiles, points showed outliers. Data points outside the whiskers can be considered as outliers. **(B)** Principal component analysis of four cattle groups. **(C)** Genome-wide average LD decay estimated from each group. **(D)** The estimation of number and length of ROH for each group.

On a genome-wide window scale of 50 kb, Yanbian cattle (East Asian taurine) showed a reduced level of nucleotide diversity compared to other groups (Figure 1A). The nucleotide diversity of Dianzhong cattle (0.0034) was second only to Chinese zebu (0.0039) and close to Indian zebu (0.0030). To explore relatedness among Dianzhong cattle and other cattle groups, PCA was conducted through using autosomal SNPs. The analysis ignored breed membership; nevertheless, it revealed clear breed structures as samples from the same group cluster together. PC1 and PC2 accordingly explained 9.12 and 4.78% of the total variations and separated taurine from indicine and Chinese zebu from Indian zebu, respectively (Figure 1B).

Demographic inferences in cattle population were made based on the analyses of ROH and LD decay. For LD patterns, at short distances, Indian zebu showed lower LD level, and the highest LD level was found in Yanbian cattle, followed by Chinese zebu and Dianzhong cattle. Yanbian cattle continued to have a higher LD than all other breeds when the distances were larger, while a contrary trend was observed in indicine (Indian zebu and Chinese zebu) and hybrid (Dianzhong) (Figure 1C). To evaluate the ROH pattern of Dianzhong cattle and other cattle groups, we divided the length of ROH into three size classes: 0.5–1, 1–2, and 2–4 Mb (Figure 1D). The vast majority of ROH identified in all groups were between 0.5 and 1 Mb in length, but apparently Yanbian cattle had more medium (1–2 Mb) and long (2–4 Mb) ROHs, showing stronger inbreeding. As expected, the pattern of ROH profile was nearly consistent with the result of LD decay.

### Candidate Regions and Genes Under Positive Selection

Due to the genetic separation between Dianzhong cattle and three reference groups, we compared Dianzhong cattle with the other three reference populations using four selective sweep methods respectively in three groups.

In the Dianzhong cattle vs. Yanbian cattle comparison, the top 1% of candidate genes in four selection methods were extracted at the intersection (Supplementary Figure S2): *MARS2* (Webb et al., 2015) and *ARL6IP6* (Abumansour et al., 2015) (growth and development), *RFTN2* (Wei et al., 2018) (DNA damage response), and *N6AMT1* (Wang et al., 2020) (feed efficiency). GO and KEGG analyses were performed on candidate genes scanned twice or more by four methods. From DAVID gene ontology, 12 significant (*p* < 0.05) GO biological process (BP) terms were enriched (Supplementary Table S16). Among them, “oxidation–reduction process, GO:0055114” (*n* = 18) contained more genes than other GO terms. The other candidate genes were mainly enriched in hot resistance (“cardiac muscle cell differentiation, GO:0055007,” “lipid catabolic process, GO:0016042,” “positive regulation of ATPase activity, GO:0032781,” “mitochondrial DNA repair, GO:0043504,” and “regulation of oxidative stress-induced intrinsic apoptotic signaling pathway, GO:1902175”). Moreover, we noticed a region of about 0.06 Mb scanned by XP-CLR on chromosome 17 (including TRNAG-CCC, *SDSL*, *SDS*, and *PLBD2*), showing a strong positive selection signal. Thereinto, a missense mutation (c.944C > A, p.Ala315Glu) was found at the *SDS* gene. This mutation presented a huge divergence between Dianzhong cattle (allele C frequency = 1) and Yanbian cattle (allele A frequency = 1).

A total of 42 candidate genes were overlapped in four selection methods by comparing Dianzhong cattle with Indian zebu (Supplementary Figure S2), and three of them were significantly enriched in “kinase binding, GO:0019900” (*p*-value = 0.02). In the functional prediction of all candidate genes, eight GO BP terms were significantly enriched (*p* < 0.05) (Supplementary Table S17). The candidate genes were mainly enriched in growth (“post-embryonic development, GO:0009791,” “skeletal muscle tissue development, GO:0007519”; and “embryonic skeletal system morphogenesis, GO:0048704”) and heat stress resistance (“DNA recombination, GO:0006310,” “inflammatory response, GO:0006954,” “response to hydrogen peroxide, GO:0042542,” and “respiratory gaseous exchange, GO:0007585”). Most genes (*n* = 11) were included in “inflammatory response, GO:0006954.” Furthermore, we detected a missense mutation (c.473A > G, p.Lys158Arg) at the fat deposition-related gene *PDGFD*. Allele A displayed a rare distribution (frequency 0.2) in Dianzhong cattle, whereas it showed an opposite pattern (frequency 0.9) in Indian zebu.

**FIGURE 2:**
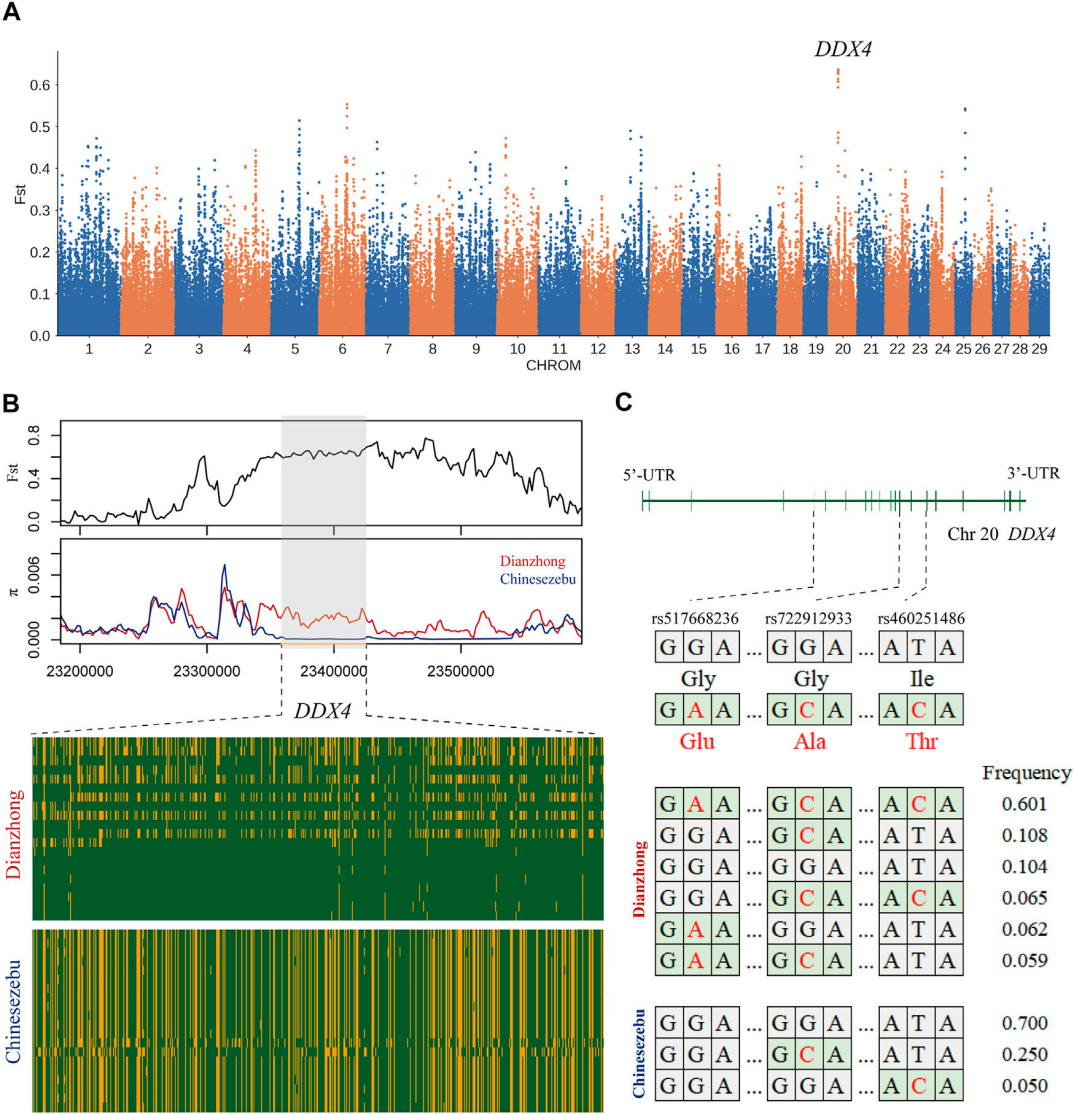
Analysis of the signatures of positive selection between Dianzhong cattle and Chinese zebu. **(A)** Manhattan plot of selective sweeps. **(B)** Fst and π plots and haplotype patterns heatmap of the DDX4 gene region. **(C)** Structure of DDX4 with exons indicated by vertical bars. Three missense mutations and their haplotypes were highlighted in red.

In the selection signal analysis between Dianzhong cattle and Chinese zebu, 36 common candidate genes were scanned by four selection methods. There were two genes enriched in the “response to retinoic acid, GO:0032526” (*p*-value = 0.04). The strongest signal was found in *KIT* gene, which was associated with sperm differentiation [33]. In KEGG analysis, four digestion-related pathways were enriched (Supplementary Table S18): “gastric acid secretion, bta04971,” “pancreatic secretion, bta04972,” “salivary secretion, bta04970,” and “carbohydrate digestion and absorption, bta04973.” In addition, three missense mutations (rs460251486, rs722912933, and rs517668236) with strong linkage effect were detected at candidate gene *DDX4*. According to the analyses for LD and haplotype construction of the three mutations, they exhibited a low recombination rate with *r* ^2^ values ranging from 0.31 to 0.61 (Supplementary Figure S3) (Ardlie et al., 2002). *DDX4* gene was the candidate gene selected by *F*st (Figure 2A), and through the calculation of *F*st and π with a smaller window (5 kb), a significant differentiation was observed between Dianzhong cattle and Chinese zebu (Figure 2B). Simultaneously, according to the calculation of the combined haplotype frequencies at the three loci, the haplotype with the most distribution in Dianzhong cattle was the homozygous mutational haplotype (frequency 0.601), while the homozygous wild haplotype (frequency 0.700) was the commonest one in Chinese zebu (Figure 2C).

## Discussion

Genomic features may reflect a variety of multifarious historical events, including climate change, species introduction, and artificial selection (Chen et al., 2021). The characteristics of population genetic diversity are essential for assessing the genetic potential of breeds as well as for the utilization and protection of cattle breed resources (Xia et al., 2021). In this study, three underlying ancestors of Dianzhong cattle were selected as reference groups to explore the genetic information of Dianzhong cattle. The nucleotide diversity ranking calculated in this study (Chinese zebu > Dianzhong cattle > Indian zebu > Yanbian cattle) was basically consistent with the earlier results (Chen et al., 2018). Dianzhong cattle, belonging to hybrid cattle, obtained more abundant genetic information from multiple genetic sources, while Chinese zebu had high nucleotide diversity which may be due to previous reception of introgression from Banteng (*Bos javanicus*) (Chen et al., 2018). The monotonous environment and high degree of breeding may be the reasons why the nucleotide diversity of Yanbian cattle is lower than that of indicine and hybrid cattle (Singh et al., 2021; Xia et al., 2021). In addition, more 2–4 MB and 1–2 MB ROHs and more rapid LD decayed in Yanbian cattle confirmed this once again. The nucleotide diversity in our Indian zebu was higher than those in other groups, and the LD level was lower than others, suggesting that Indian zebu had a higher effective population size (Chen et al., 2021). In summary, the LD decay pattern and ROH distribution of each group were roughly consistent with the results of nucleotide diversity.

Owing to the humid and hot living environment, Dianzhong cattle evolved to be prominently heat resistant (China National Commission of Animal Genetic Resources, 2011). In the comparative analysis with Yanbian cattle living in a cold environment, functional enrichment analysis revealed that heat resistance-related pathways were significantly overrepresented in candidate genes under positive selection. Moreover, a putatively selected gene, *SDS*, encodes serine dehydratase, an enzyme that catalyzes the degradation of serine to pyruvate, which was functionally involved in heat resistance (Ogawa et al., 1989). *SDS* activated inflammasomes by mediating mitochondrial membrane potential, thereby affecting individual immune function (Çağdaş et al., 2020). Inflammasomes are important in the defense against microbial infections and have roles in shaping the adaptive immune responses (Joly et al., 2012; Yu et al., 2015).

The typical characteristic of Dianzhong cattle is small in body size, approximately 101 cm tall and weighing 170 kg in adulthood (Wen et al., 2014). Compared with the tall Indian zebu by selection analysis, there were many growth- and development-related items in GO enrichment. What is more, because of the preference of East Asians for good meat quality (high marbling score), Dianzhong cattle have high potential for accumulating intramuscular fat and producing highly marbled beef (Hao et al., 2017). The *PDGFD* gene selected by XP-CLR was related to perivascular adipose tissue generation (Dong et al., 2020). Members of the PDGF family have an anti-fat effect and inhibit the differentiation of preadipocytes (Ma et al., 2018; Pan et al., 2019). *PDGFD* played a pivotal role in inhibiting the differentiation of white adipocytes by regulating the expression of PPARγ2 and C/EBPα (Li et al., 2020). Early studies have reported that the *PDGFD* gene was hypothesized to be one of the candidate genes leading to fat tail formation in indigenous sheep (Zhao et al., 2020; Zhu et al., 2021). In addition to the possible involvement of intermuscular fat deposition, we ventured to speculate that the *PDGFD* gene may also contribute to the formation of indicine hump. However, this is just a conjecture, and more theoretical and experimental supports were required.

Poor fertility is one of the most important factors restricting the development of Dianzhong cattle (China National Commission of Animal Genetic Resources, 2011). The process of admixture among the *Bos* subspecies brought adaptability but caused a cost of reduced reproductive fitness due to genomic incompatibility at the same time (Kim et al., 2020). Jiangxi cattle (Chinese zebu) is a precocious puberty cattle breed that can breed for the first time at the age of 10 months (Shuanping et al., 2019), while Dianzhong bulls do so later than 30 months old (China National Commission of Animal Genetic Resources, 2011). In comparison between Dianzhong cattle and Chinese zebu, some candidate genes were significantly enriched in “response to retinoic acid, GO:0032526” (*p* = 0.04). Retinoic acid promoted the differentiation of male germ cells and induced the differentiation of mouse pluripotent cell bodies into outer male germ cells (Mahabadi et al., 2020). In addition, candidate gene *KIT*, as the highest-ranked in overlap, is a transmembrane protein receptor related to germ cell maturation (Rossi et al., 2000). It is also a sign of the loss of efficacy of spermatogonial stem cells (Schrans-Stassen et al., 1999). In general, the presence of multiple linkage loci associated with a specific phenotype in a gene indicated a highly probable connection between the gene and this phenotype (Jia et al., 2019; Cao et al., 2020). Hence, the haplotypes formed by the three strongly linked missense mutations in the *DDX4* gene were obviously different between Dianzhong cattle and Chinese zebu, implying that the spermatogenesis-related *DDX4* gene may be related to the weak reproductive performance of Dianzhong cattle. *DDX4* gene encodes an ATP-dependent RNA helicase (Hay et al., 1988). In *DDX4*-knockout mice, germ cell development was normal in female homozygous null mice, but male mice were sterile due to the failure of their germ cells to progress from leptotene to zygotene of meiotic prophase I, and the cells underwent apoptosis (Tanaka et al., 2000). The expression of *DDX4* in germ cells of male mice has been described in humans, dogs, cattle, pigs, and stallions (Lee et al., 2018a). Interestingly, *KIT* gene, together with *DDX4* gene, was considered to be a marker for undifferentiated spermatogonia before puberty and differentiated spermatogonia after puberty in porcine testis (Lee et al., 2018b).

In conclusion, our population genomic analyses of Dianzhong cattle and other three reference groups provide novel insights into their genetic diversity and selective sweep. This will point out the direction for genetic assessment and development of reasonable improvement of Dianzhong cattle. Moreover, we identified a series of candidate genes that may be important for the heat resistance, intermuscular fat deposition, and reproductive barriers of this breed. These results provide a basis for further research on the genome characteristics of other important indigenous beef cattle in the future (Gao et al., 2017).

## Data Availability

The datasets presented in this study can be found in online repositories. The names of the repository/repositories and accession number(s) can be found in the article/Supplementary Material.

## References

[B1] Abumansour I. S. Hijazi H. Alazmi A. Alzahrani F. Bashiri F. A. Hassan H. (2015). ARL6IP6, a Susceptibility Locus for Ischemic Stroke, Is Mutated in a Patient with Syndromic Cutis Marmorata Telangiectatica Congenita. Hum. Genet. 134 (8), 815–822. 10.1007/s00439-015-1561-6 25957586

[B2] Ardlie K. G. Kruglyak L. Seielstad M. (2002). Patterns of Linkage Disequilibrium in the Human Genome. Nat. Rev. Genet. 3 (4), 299–309. 10.1038/nrg777 11967554

[B3] Bhati M. Kadri N. K. Crysnanto D. Pausch H. (2020). Assessing Genomic Diversity and Signatures of Selection in Original Braunvieh Cattle Using Whole-Genome Sequencing Data. BMC Genomics 21 (1), 27. 10.1186/s12864-020-6446-y 31914939 PMC6950892

[B4] Bolger A. M. Lohse M. Usadel B. (2014). Trimmomatic: A Flexible Trimmer for Illumina Sequence Data. Bioinformatics (Oxford, England) 30 (15), 2114–2120. 10.1093/bioinformatics/btu170 PMC4103590 24695404

[B5] Bovine H. C. Gibbs R. A. Taylor J. F. Van Tassell C. P. Barendse W. Eversole K. A. (2009). Genome-wide Survey of SNP Variation Uncovers the Genetic Structure of Cattle Breeds. Science 324 (5926), 528–532. 10.1126/science.1167936 19390050 PMC2735092

[B6] Cao Y. Jia P. Wu Z. Huang M. Chen S. Zhang J. (2020). A Novel SNP of MYO1A Gene Associated with Heat-Tolerance in Chinese Cattle. Anim. Biotechnol. 4, 1–6. 10.1080/10495398.2020.1837147 33146068

[B7] Çağdaş D. Sürücü N. Tan Ç. Kayaoğlu B. Özgül R. K. Akkaya-Ulum Y. Z. (2020). Autoinflammation in Addition to Combined Immunodeficiency: SLC29A3 Gene Defect. Mol. Immunol. 121, 28–37. 10.1016/j.molimm.2020.02.014 32151906

[B8] Chen N. Cai Y. Chen Q. Li R. Wang K. Huang Y. (2018). Whole-genome Resequencing Reveals World-wide Ancestry and Adaptive Introgression Events of Domesticated Cattle in East Asia. Nat. Commun. 9 (1), 2337. 10.1038/s41467-018-04737-0 29904051 PMC6002414

[B9] Chen Q. Shen J. Hanif Q. Chen N. Huang Y. Dang R. (2021). Whole Genome Analyses Revealed Genomic Difference between European Taurine and East Asian Taurine. J. Anim. Breed. Genet. 138 (1), 56–68. 10.1111/jbg.12501 32770713

[B10] China National Commission of Animal Genetic Resources (2011). Animal Genetic Resources in China Bovines. Beijing: Chinese Agricultural Press.

[B11] Danecek P. Auton A. Abecasis G. Albers C. A. Banks E. DePristo M. A. (2011). The Variant Call Format and VCFtools. Bioinformatics 27 (15), 2156–2158. 10.1093/bioinformatics/btr330 21653522 PMC3137218

[B12] Dong K. Yang M. Han J. Ma Q. Han J. Song Z. (2020). Genomic Analysis of Worldwide Sheep Breeds Reveals PDGFD as a Major Target of Fat-Tail Selection in Sheep. BMC Genomics 21 (1), 800. 10.1186/s12864-020-07210-9 33203382 PMC7670677

[B13] Gao Y. Gautier M. Ding X. Zhang H. Wang Y. Wang X. (2017). Species Composition and Environmental Adaptation of Indigenous Chinese Cattle. Sci. Rep. 7 (1), 16196. 10.1038/s41598-017-16438-7 29170422 PMC5700937

[B14] Gibson J. Morton N. E. Collins A. (2006). Extended Tracts of Homozygosity in Outbred Human Populations. Hum. Mol. Genet. 15 (5), 789–795. 10.1093/hmg/ddi493 16436455

[B15] Hao W. Zhixian X. Peichang Y. Chunfeng L. Xiangsheng S. Rongfa L. (2017). Protection, Exploitation and Utilization of Genetic Resources in Dianzhong Cattle. Curr. Anim. husbandry 403 (35), 6–7.

[B16] Hay B. Jan L. Y. Jan Y. N. (1988). A Protein Component of Drosophila Polar Granules Is Encoded by Vasa and Has Extensive Sequence Similarity to ATP-dependent Helicases. Cell 55 (4), 577–587. 10.1016/0092-8674(88)90216-4 3052853

[B17] Huang D. W. Sherman B. T. Lempicki R. A. (2009). Systematic and Integrative Analysis of Large Gene Lists Using DAVID Bioinformatics Resources. Nat. Protoc. 4 (1), 44–57. 10.1038/nprot.2008.211 19131956

[B18] Jia P. Cai C. Qu K. Chen N. Jia Y. Hanif Q. (2019). Four Novel SNPs of MYO1A Gene Associated with Heat-Tolerance in Chinese Cattle. Animals 9 (11), 964. 10.3390/ani9110964 PMC6912737 31766183

[B19] Joly S. Eisenbarth S. C. Olivier A. K. Williams A. Kaplan D. H. Cassel S. L. (2012). Cutting Edge: Nlrp10 Is Essential for Protective Antifungal Adaptive Immunity against Candida Albicans, J Immunol, 10, 189. Baltimore, Md. *1950* , 4713–7. 10.4049/jimmunol.1201715 23071280 PMC3548226

[B20] Kim J. Hanotte O. Mwai O. A. Dessie T. Bashir S. Diallo B. (2017). The Genome Landscape of Indigenous African Cattle. Genome Biol. 18 (1), 34. 10.1186/s13059-017-1153-y 28219390 PMC5319050

[B21] Kim K. Kwon T. Dessie T. Yoo D. Mwai O. A. Jang J. (2020). The Mosaic Genome of Indigenous African Cattle as a Unique Genetic Resource for African Pastoralism. Nat. Genet. 52 (10), 1099–1110. 10.1038/s41588-020-0694-2 32989325

[B22] Lee R. Lee W.-Y. Park H.-J. Ha W.-T. Woo J.-S. Chung H.-J. (2018a). Stage-specific Expression of DDX4 and C-Kit at Different Developmental Stages of the Porcine Testis. Anim. Reprod. Sci. 190, 18–26. 10.1016/j.anireprosci.2017.12.020 29338902

[B23] Lee R. Lee W.-Y. Park H.-J. Ha W.-T. Woo J.-S. Chung H.-J. Lee J.-H. Hong K. Song H. (2018b). Stage-specific expression of DDX4 and c-kit at different developmental stages of the porcine testis. Animal Reproduction Science 190, 18–26. 10.1016/j.anireprosci.2017.12.020 29338902

[B24] Li H. Durbin R. (2009). Fast and Accurate Short Read Alignment with Burrows-Wheeler Transform. Bioinformatics 25 (14), 1754–1760. 10.1093/bioinformatics/btp324 19451168 PMC2705234

[B25] Li R. Li C. Chen H. Liu X. Xiao H. Chen S. (2019). Genomic Diversity and Admixture Patterns Among Six Chinese Indigenous Cattle Breeds in Yunnan. Asian-australas J. Anim. Sci. 32 (8), 1069–1076. 10.5713/ajas.18.0605 30744361 PMC6599958

[B26] Li X. Yang J. Shen M. Xie X.-L. Liu G.-J. Xu Y.-X. (2020). Whole-genome Resequencing of Wild and Domestic Sheep Identifies Genes Associated with Morphological and Agronomic Traits. Nat. Commun. 11 (1), 2815. 10.1038/s41467-020-16485-1 32499537 PMC7272655

[B27] Ma L. Li Z. Cai Y. Xu H. Yang R. Lan X. (2018). Genetic Variants in Fat- and Short-Tailed Sheep from High-Throughput RNA-Sequencing Data. Anim. Genet. 49 (5), 483–487. 10.1111/age.12699 30069889

[B28] Mahabadi J. A. Tameh A. A. Talaei S. A. Karimian M. Rahiminia T. Enderami S. E. (2020). Retinoic Acid And/or Progesterone Differentiate Mouse Induced Pluripotent Stem Cells into Male Germ Cells *In Vitro* . J. Cel. Biochem. 121 (3), 2159–2169. 10.1002/jcb.29439 31646671

[B29] Nekrutenko A. Taylor J. (2012). Next-generation Sequencing Data Interpretation: Enhancing Reproducibility and Accessibility. Nat. Rev. Genet. 13 (9), 667–672. 10.1038/nrg3305 22898652

[B30] Ogawa H. Gomi T. Konishi K. Date T. Nakashima H. Nose K. (1989). Human Liver Serine Dehydratase. J. Biol. Chem. 264 (27), 15818–15823. 10.1016/S0021-9258(18)71550-0 2674117

[B31] Pan Z. Li S. Liu Q. Wang Z. Zhou Z. Di R. (2019). Rapid Evolution of a Retro-Transposable Hotspot of Ovine Genome Underlies the Alteration of BMP2 Expression and Development of Fat Tails. BMC Genomics 20 (1), 261. 10.1186/s12864-019-5620-6 30940097 PMC6445056

[B32] Patterson N. Price A. L. Reich D. (2006). Population Structure and Eigenanalysis. Plos Genet. 2, e190–e2093. 10.1371/journal.pgen.0020190 17194218 PMC1713260

[B33] Purcell S. Neale B. Todd-Brown K. Thomas L. Ferreira M. A. R. Bender D. (2007). PLINK: A Tool Set for Whole-Genome Association and Population-Based Linkage Analyses. Am. J. Hum. Genet. 81 (3), 559–575. 10.1086/519795 17701901 PMC1950838

[B34] Rossi P. Sette C. Dolci S. Geremia R. (2000). Role of C-Kit in Mammalian Spermatogenesis. J. Endocrinol. Invest. 23 (9), 609–615. 10.1007/BF03343784 11079457

[B35] Schrans-Stassen B. H. G. J. van de Kant H. J. G. de Rooij D. G. van Pelt A. M. M. (1999). Differential Expression of C-Kit in Mouse Undifferentiated and Differentiating Type a Spermatogonia. Endocrinology 140 (12), 5894–5900. 10.1210/endo.140.12.7172 10579355

[B36] Shen J. Hanif Q. Cao Y. Yu Y. Lei C. Zhang G. (2020). Whole Genome Scan and Selection Signatures for Climate Adaption in Yanbian Cattle. Front. Genet. 11, 94. 10.3389/fgene.2020.00094 32180793 PMC7059643

[B37] Shuanping Z. Hai J. Lei X. Juan W. Mo L. Yutang J. (2019). Analysis on the Characteristics of Wannan Cattle Germplasm Resources and Hybridization Improvement Effect. Chin. Cattle Sci. 45 (05), 21–23.

[B38] Singh A. K. Liu W. Zakari S. Wu J. Yang B. Jiang X. J. (2021). A Global Review of Rubber Plantations: Impacts on Ecosystem Functions, Mitigations, Future Directions, and Policies for Sustainable Cultivation. Sci. Total Environ. 796, 148948. 10.1016/j.scitotenv.2021.148948 34273842

[B39] Stothard P. Choi J.-W. Basu U. Sumner-Thomson J. M. Meng Y. Liao X. (2011). Whole Genome Resequencing of Black Angus and Holstein Cattle for SNP and CNV Discovery. BMC Genomics 12, 559. 10.1186/1471-2164-12-559 22085807 PMC3229636

[B40] Sun T. Huang G.-y. Wang Z.-h. Teng S.-h. Cao Y.-h. Sun J.-l. (2020). Selection Signatures of Fuzhong Buffalo Based on Whole-Genome Sequences. BMC Genomics 21 (1), 674. 10.1186/s12864-020-07095-8 32993537 PMC7526191

[B41] Tanaka S. S. Toyooka Y. Akasu R. Katoh-Fukui Y. Nakahara Y. Suzuki R. (2000). The Mouse Homolog of Drosophila Vasa Is Required for the Development of Male Germ Cells. Genes Dev. 14 (7), 841–853. 10.1101/gad.14.7.841 10766740 PMC316497

[B42] Taye M. Kim J. Yoon S. H. Lee W. Hanotte O. Dessie T. (2017). Whole Genome Scan Reveals the Genetic Signature of African Ankole Cattle Breed and Potential for Higher Quality Beef. BMC Genet. 18 (1), 11. 10.1186/s12863-016-0467-1 28183280 PMC5301378

[B43] Utsunomiya Y. T. Milanesi M. Fortes M. R. S. Porto‐Neto L. R. Utsunomiya A. T. H. Silva M. V. G. B. (2019). Genomic Clues of the Evolutionary History of *Bos indicus* Cattle. Anim. Genet. 50 (6), 557–568. 10.1111/age.12836 31475748

[B44] Wang K. Li M. Hakonarson H. (2010). ANNOVAR: Functional Annotation of Genetic Variants from High-Throughput Sequencing Data. Nucleic Acids Res. 38 (16), e164. 10.1093/nar/gkq603 20601685 PMC2938201

[B45] Wang X. Kadarmideen H. N. (2020). Metabolite Genome-wide Association Study (mGWAS) and Gene-Metabolite Interaction Network Analysis Reveal Potential Biomarkers for Feed Efficiency in Pigs. Metabolites 10 (5), 201. 10.3390/metabo10050201 PMC7281523 32429265

[B46] Webb B. D. Wheeler P. G. Hagen J. J. Cohen N. Linderman M. D. Diaz G. A. (2015). Novel, Compound Heterozygous, Single-Nucleotide Variants inMARS2Associated with Developmental Delay, Poor Growth, and Sensorineural Hearing Loss. Hum. Mutat. 36 (6), 587–592. 10.1002/humu.22781 25754315 PMC4439286

[B47] Wei F. Hao P. Zhang X. Hu H. Jiang D. Yin A. (2018). Etoposide-induced DNA Damage Affects Multiple Cellular Pathways in Addition to DNA Damage Response. Oncotarget 9 (35), 24122–24139. 10.18632/oncotarget.24517 29844877 PMC5963631

[B48] Wen L. Deliang C. Xiaofang O. Jincheng Z. Xiangguang M. Jiacai Z. (2014). Determination and Analysis of Growth Performance of Hybrid Progeny of Dianzhong Cattle. Shanghai Anim. husbandry Vet. Newsl. 01, 32–35.

[B49] Wu D.-D. Ding X.-D. Wang S. Wójcik J. M. Zhang Y. Tokarska M. (2018). Pervasive Introgression Facilitated Domestication and Adaptation in the Bos Species Complex. Nat. Ecol. Evol. 2 (7), 1139–1145. 10.1038/s41559-018-0562-y 29784979

[B50] Xia X. Zhang S. Zhang H. Zhang Z. Chen N. Li Z. (2021). Assessing Genomic Diversity and Signatures of Selection in Jiaxian Red Cattle Using Whole-Genome Sequencing Data. BMC Genomics 22 (1), 43. 10.1186/s12864-020-07340-0 33421990 PMC7796570

[B51] Yong Y. He L. (2005). SHEsis, a Powerful Software Platform for Analyses of Linkage Disequilibrium, Haplotype Construction, and Genetic Association at Polymorphism Loci. Cell Res 15 (2), 97–98. 10.1038/sj.cr.7290272 15740637

[B52] Yu S.-X. Du C.-T. Chen W. Lei Q.-Q. Li N. Qi S. (2015). Genipin Inhibits NLRP3 and NLRC4 Inflammasome Activation via Autophagy Suppression. Sci. Rep. 5, 17935. 10.1038/srep17935 26659006 PMC4675967

[B53] Zhang C. Dong S.-S. Xu J.-Y. He W.-M. Yang T.-L. (2019). PopLDdecay: A Fast and Effective Tool for Linkage Disequilibrium Decay Analysis Based on Variant Call Format Files. Bioinformatics 35 (10), 1786–1788. 10.1093/bioinformatics/bty875 30321304

[B54] Zhao F. Deng T. Shi L. Wang W. Zhang Q. Du L. (2020). Genomic Scan for Selection Signature Reveals Fat Deposition in Chinese Indigenous Sheep with Extreme Tail Types. Animals 10 (5), 773. 10.3390/ani10050773 PMC7278473 32365604

[B55] Zhu C. Li N. Cheng H. Ma Y. (2021). Genome Wide Association Study for the Identification of Genes Associated with Tail Fat Deposition in Chinese Sheep Breeds. Biol. Open 10 (5). 10.1242/bio.054932 PMC8186729 33942864

