# Supplementary material for: Assessing Genomic Diversity and Productivity Signatures in Dianzhong Cattle by Whole-Genome Scanning
Source: Front Genet. 2021 Oct 5;12:719215. doi: 10.3389/fgene.2021.719215 (PMC8523829; doi:10.3389/fgene.2021.719215)
Supplement: Supplementary file 1 [file DataSheet2.PDF]

(**A**)

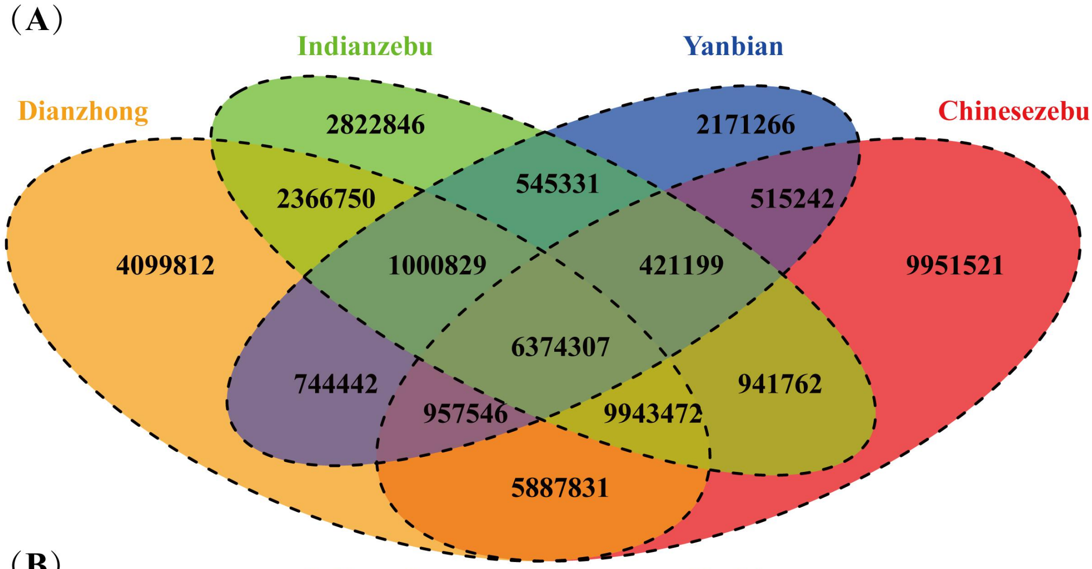

(**B**)

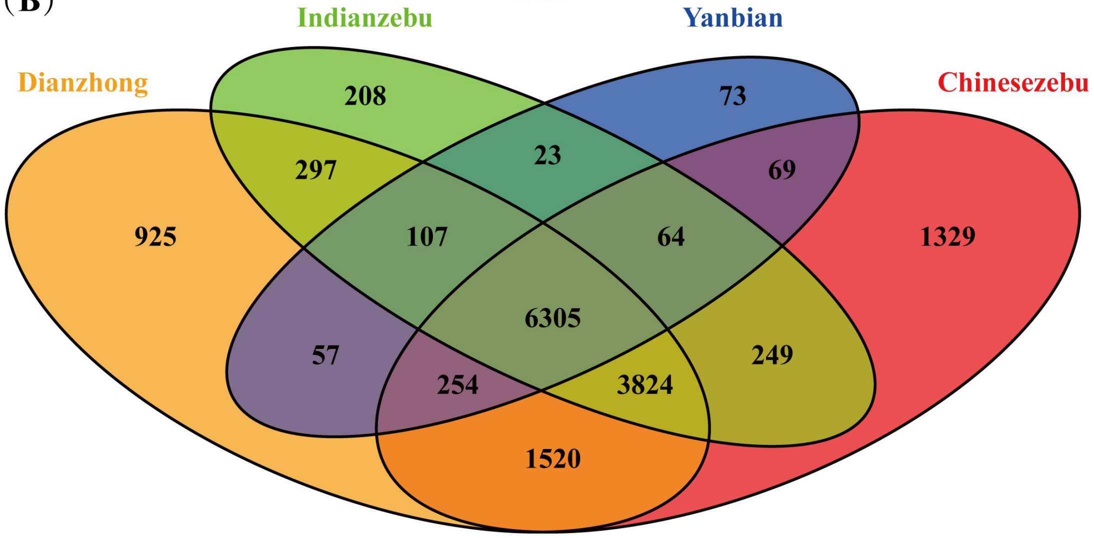

Supplementary Figure 1. Venn diagram showing overlapping and unique SNPs among different groups. (A) The unique and shared SNPs, (B) The non-synonymous SNPs.
