# Supplementary material for: Assessing Genomic Diversity and Productivity Signatures in Dianzhong Cattle by Whole-Genome Scanning
Source: Front Genet. 2021 Oct 5;12:719215. doi: 10.3389/fgene.2021.719215 (PMC8523829; doi:10.3389/fgene.2021.719215)
Supplement: Supplementary file 3 [file DataSheet3.PDF]

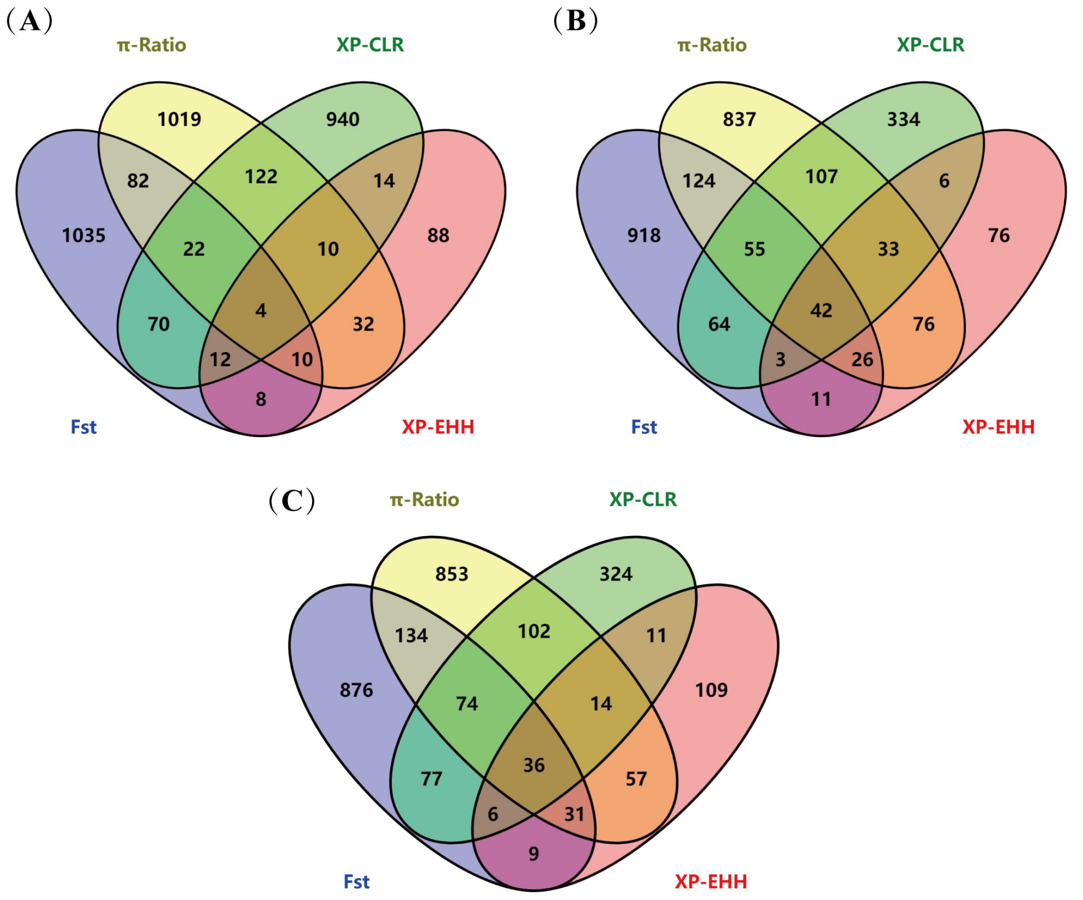

Supplementary Figure 2. Overlap of four selection methods in three groups. Dianzhong cattle was compared with (A) Yanbian cattle, (B) Indianzebu and (C) Chinesezebu.
