# Supplementary material for: Assessing Genomic Diversity and Productivity Signatures in Dianzhong Cattle by Whole-Genome Scanning
Source: Front Genet. 2021 Oct 5;12:719215. doi: 10.3389/fgene.2021.719215 (PMC8523829; doi:10.3389/fgene.2021.719215)
Supplement: Supplementary file 4 [file DataSheet1.PDF]

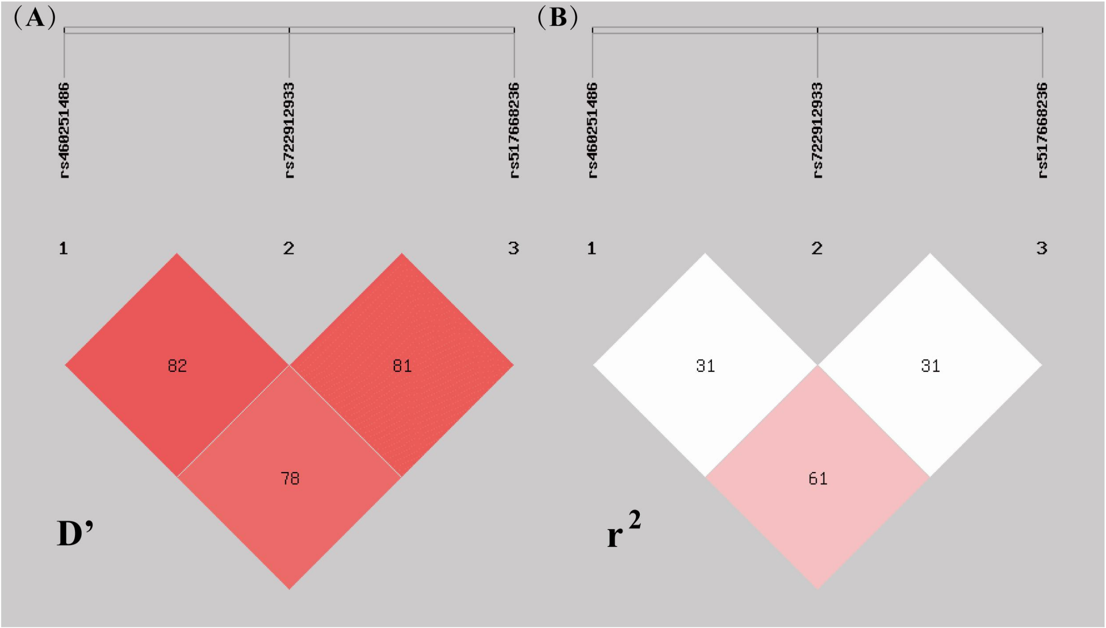

Supplementary Figure 3. Linkage disequilibrium analysis of three missense mutations in the *DDX4* gene. (A) D′ value of three missense mutations, (B) r^2 value of three missense mutations.
